# Therapeutic lying: Brazilian speech and language therapists’ point of view about a controversial communication strategy employed in the care for people with dementia

**DOI:** 10.1590/2317-1782/20212021252en

**Published:** 2022-10-21

**Authors:** Isabella Amaral Lopes, Emily Viega Alves, Bárbara Costa Beber

**Affiliations:** 1 Curso de Fonoaudiologia, Universidade Federal de Ciências da Saúde de Porto Alegre – UFCSPA - Porto Alegre (RS), Brasil.; 2 Departamento de Fonoaudiologia, Universidade Federal de Ciências da Saúde de Porto Alegre – UFCSPA - Porto Alegre (RS), Brasil.; 3 Programa de Pós-graduação em Ciências da Reabilitação, Universidade Federal de Ciências da Saúde de Porto Alegre – UFCSPA - Porto Alegre (RS), Brasil.

**Keywords:** Dementia, Communication, Speech-Language and Hearing Sciences, Deception, Communication Barriers

## Abstract

**Purpose:**

The objective of this research was to obtain the speech and language therapists’ point of view about the use of therapeutic lying as a communication strategy in dementia care.

**Methods:**

The present research was a quantitative, qualitative, and descriptive cross-sectional study. Data was collected through an online survey with multiple choices and open answer questions.

**Results:**

The quantitative results indicated that the majority of the speech and language therapists have already used therapeutic lying as a communicative strategy and wish to learn more about it, considering the technique as relatively valid, ethical and adequate. The qualitative results indicated the reasons for the usage of the technique: to reassure the patient in case of agitation; to encourage engagement in therapy; to avoid stress-related to memory loss; to manage difficulty or refusal to eat; to manage difficulty or refusal for drug treatment; to prevent patients from leaving the building; to manage delirium, confusion and/or paranoia; to ensure safety; and for use when other strategies do not work.

**Conclusion:**

The majority of speech and language therapists use therapeutic lying in their clinical practice, taking into consideration the best interest of the person with dementia, although professionals recognize their lack of knowledge on the subject. They have considered the communication strategy as relatively ethical, valid and adequate. The article calls attention to the necessity of education and guidelines for speech and language therapists in the use of therapeutic lying among people with dementia.

## INTRODUCTION

Dementia is a neurological disorder characterised by cognitive and functional decline, in which there is loss in two or more cognitive domains (such as memory, executive functions, attention, language, social behaviour and judgement, psychomotor abilities, or others) and it is not related to natural aging^(1)^. It is defined as an acquired syndrome, usually of a chronic or progressive nature, that significantly impairs the independence of the affected person^(2)^.

Data from the Pan American Health Organisation^(3)^ indicates that the number of people with dementia may triple from 50 million to 152 million by 2050, due to world population aging. In Brazil, many studies^(4,5)^ show dementia prevalence by regions with variable results. The most common types of dementia are Alzheimer's disease (AD), vascular dementia, Lewy body dementia, Parkinson's disease, and frontotemporal dementia^(4,5)^.

People with dementia often have behavioural changes that are difficult to deal with. These changes are called challenging behaviours^(6)^, which impairs the lives of this population physically, psychologically, socially and economically. Studies estimate that more than 90% of people with dementia develop at least one challenging behaviour^(7)^, which maybe a predictor for the admission of this population into long-term care facilities^(8)^. Furthermore, people with dementia also present language impairment, which can cause difficulty to keep a conversation^(9)^, besides the impairments in memory and other cognitive abilities. In this context, Speech and Language Therapists (SLTs) have an important role in the rehabilitation of this population since they facilitate adaptation and training of cognitive abilities, optimization of communicative and behavioural functions, and make use of compensatory communication strategies tailored to each individual^(10)^.

Communication skills are essential for health professionals who work with patients under palliative care, regardless of their training or area of expertise^(11)^. One of the most frequently used communication strategies to deal with challenging behaviour^(12-15)^ is Therapeutic Lying (TL). This term refers to lying that aims to serve the patient's interest^(12)^, especially when there is a disagreement between the reality of the caregiver/health professional and the person with dementia^(16,17)^. Studies show that most health professionals have already used TL in clinical practice^(6,13,18)^. TL can take on a quick-fix role in dealing with critical moments and can be seen as a non-pharmacological intervention in the management of challenging behaviour^(12)^. In addition, it can be used by SLTs as a communication tool in cases when the person with dementia is unable to understand the information received or the action requested, and of them making decisions that are in their best interest.

What makes this communication strategy controversial is the fact that according to Matias et al.^(19)^, lying is characterised as the act of “trying to convince another person to accept something that is false for one's benefit or that of another, to maximise a gain or avoid a loss”, which creates a negative social value as it is usually used to manipulate, deceive, or distract another person^(13)^. These characteristics bring up the question of TL being in agreement with moral and ethics values, mainly in the professional field. There is the possibility that TL may be considered a form of abuse of the autonomy of the person with dementia if it is not regulated or is used inappropriately or ineffectively. For this reason, it is necessary to know when the person with dementia is able to decide by her/himself and also how to use this strategy in an efficient way^(12)^.

When considering TL as a communication strategy, the SLTs, who are health professionals and work in all aspects related to human communication, should play a fundamental role in guidance for the use of TL by caregivers or other health professionals. However, there are no reports in the literature about SLTs' practices nor research on this topic. To make it possible for SLTs to contribute to the guidance for the use of TL it is firstly necessary to know if they know about this communication strategy and what is their point of view about the use of it. Despite getting more attention recently^(6,20-22)^, there are still few studies on the use of TL as a communication strategy in the care of people with dementia, even though it is frequently used in clinical practice. It is essential to inform SLTs about the technique of TL and qualify them to step in appropriately in situations in which patients present challenging behaviour. In this way, the purpose of the present study is to obtain the opinion of Brazilian SLTs regarding the use of TL as a communication strategy in the management of people with dementia.

## METHODS

This is a quantitative and qualitative, cross-sectional and descriptive research, which has been approved by the local ethics committee under protocol number 2.733.408. The sample of this study is characterised as a convenience sample.

Data collection was carried out through an online questionnaire (Annex A) created by the researchers. The questionnaire had 25 questions, 14 of which were multiple choice questions and 11 were open answer questions. The questionnaire was made available on Google Forms platform and was aimed at Brazilian SLTs. Access to the questions was only allowed after SLTs read and agreed with the consent form. To consent to participate in the study, the participant needed to click “I agree”.

The study was disseminated through social media and email lists. This was exclusively for SLTs born and working in Brazil with experience in the area of dementia. SLT students, retired or non-active SLTs in the specific area were excluded. The participants who did not complete the questionnaire were also excluded from the study.

Data was exported from Google Forms to Google SpreadSheet and transformed into Microsoft Excel to be analysed later using the Statistical Package for the Social Sciences Program (SPSS), version 21. Regarding the quantitative data, continuous variables were described on mean and standard deviation, and categorical variables in absolute and relative frequency. Qualitative data was analysed using the content analysis method^(23)^, summarised by the authors and described in a narrative form.

## RESULTS

### Quantitative data

82 individuals answered the questionnaire. One was excluded for not being a SLT and nine for not having experience with people with dementia. Therefore, the final sample consisted of 72 participants.

Regarding the descriptive data, female participants made up 100% of the sample. The most frequent educational level was postgraduation (48.6%), followed by PhD (18.1%), master's degree (13.9%), undergraduation (12.5%) and post-doc (6.9%). The Southern region of Brazil was the most frequent demographic region (45.8%), followed by the Southeast (33.3%), Midwest (12.5%), Northeast (6.9%) and North (1.4%). The study time for SLTs averaged 1.51 years (±9.89) and the time of experience with dementia was 7.56 years (±5.97).

Concerning the opinion of SLTs about the use of TL, 77.8% considered the practice relatively ethical, 12.5% considered it very ethical and 9.7% did not consider it ethical at all. When they were asked how valid is it to lie to benefit the patient and reduce caregivers' stress, 65.3% considered it relatively valid, while 27.8% considered it very valid and 6.9% not valid at all. Finally, regarding the opinion of the sample on the use of TL by the professional class, 68.1% expressed that the practice is relatively appropriate, 23.6% agreed it is very appropriate and 8.3% considered that it is not appropriate at all.

Table 1 shows the use and knowledge of TL by SLTs participants in the study and Table 2 represents situations in which TL could be used from the participants' point of view.

**Table 1:** Use and knowledge on TL by SLTs participants in the study (frequency corresponds to “yes” answers)

	**N (%)**
Have you ever lied to a patient with dementia to benefit her/him?	47 (65.3)
Have you ever guided relatives or caregivers of people with dementia on the use of TL as a communication strategy?	35 (48.6)
Had you ever heard about the term TL before this research?	17 (23.6)
Have you ever found bibliographic references on TL?	4 (5.6)
Have you been taught about TL during your training?	6 (8.3)
Would you like to learn more about TL?	59 (81.9)

Caption: TL = therapeutic lying

**Table 2:** Situations in which therapeutic lying could be used

	N (%)
To convince the patient to take the medications or receive treatments/health follow-ups	53 (82.8)
To convince the patient to perform personal hygiene	46 (71.9)
To convince the patient to not leave the house	40 (62.5)
To convince the patient to eat well	51 (79.7)
In no situation	7 (10.9)
Others	10 (15.6)

### Qualitative data

For qualitative data, open answer questions were created for participants to comment on their choices regarding the reasons and situations that led some of them to use TL as a strategy in their clinical practice. To perform the analysis of this data, the content analysis method^(23)^ was used. Some examples of participants' reports are shown in Chart 1.

**Chart 1:** Qualitative data

Reasons for using TL	Examples of reports
To calm down the dementia sufferer in a perturbed situation	Professional informed the patient that her/his companion was in the waiting room, even though they war not, just to reassure them.
Collaboration with the therapy	SLT mentioned that the patient's appointment was paid by the health plan, when in fact the family paid for it.
To avoid stress related to memory loss	TL was used to avoid suffering of patients who did not remember the death of their spouses or loved ones.
Difficulty or refusal to eat	A professional reported that a patient only accepted the diet when he was told that it had been prepared by his mother or aunt, both dead.
Difficulty or refusal to take medication	A professional reported having working together with a doctor to find ways to minimise stress in medication administration.
To prevent the patient from escaping the place	TL used to prevent the patient from becoming frustrated or perturbed due to the impossibility of being discharged.
To deal with confusion and/or paranoia	SLT has used TL to confirm that she was a nurse and would heal the patient's war wounds.
To ensure safety	A professional experienced a patient who did not walk and wanted to get up from her chair to leave the house and used TL to discourage her from doing this.
When other strategies do not work	TL has been the only effective resource when changing the patient's focus of attention was not effective.

Concerning how valid and ethical the use of TL would be, the answers could be responded to according to a Likert scale (not at all, relatively and very valid or ethical).

When the participants were asked how valid they believed it to be to lie to the patient to benefit them, those who considered the practice “very valid” thought that TL rapidly ensures the patients cooperation, tranquillity and their safety. The SLTs who defended this option, reinforced the importance of family authorization. The individuals who chose to answer “relatively valid”, affirmed that it is necessary to investigate the appropriate moment for the use of TL and the content used. These professionals considered using TL as a last resource, first applying other strategies, such as the use of distracting themes to change focus. The SLTs who marked the option “not valid at all” believed that lying constitutes a mistake and hurts interpersonal relationships. They thought that the benefits for the use of TL would be more aligned to family members and caregivers. In this way, they believe that other techniques could play a better role than the use of lying.

Regarding how ethical the individuals consider TL, those who marked the option “very ethical” claimed that the intention of the TL is not to deceive or hide something from the patient, but to manage the behaviour he/she presents for his/her own good. The participants who considered the use of TL as “relatively ethical”, explained their choice through the importance of keeping patient's autonomy and the quality of the information used in the strategy. They also considered family's participation fundamental. When selecting the option “not at all ethical”, the motivation presented by the participants was mainly based on the definition of ethics, since for them, lying characterises a disruption of this principle.

With regards to comments on other situations in which TL could be used, only answers that had not been previously mentioned in other items of the questionnaire were considered. The only issue highlighted in this section was the need for knowledge about TL as a communication strategy.

## DISCUSSION

The present study has investigated the view of Brazilian SLTs on the use of TL as a communication strategy in the management of individuals with dementia, since the profession strategically studies human communication. The SLT's role in this issue of TL could be through the use of TL as a communication strategy in the care of people with dementia. It is used in order to decrease behavioural changes in patients and ensure better participation of them in therapies. In addition, SLTs can train caregivers and others health professionals on how to use TL properly with people suffering with dementia.

The findings of this study confirm the results of research previously carried out by other health professionals, such as psychologists, psychiatrists and nurses^(12,15)^ in relation to the fact that these professionals had already used TL in their clinical practice, irrespective if they had not been properly informed about this communication strategy and its ethical issues.

Chart 1 highlights the various situations in which participants used TL. It was evident that in all situations, the objective was to work for the best interests of the patient. Among the different categories exposed, attention was drawn to the topic “difficulty or refusal to eat”. According to the Brazilian Resolution of the Federal Council of Speech-Language Therapy and Audiology, nº 492, of April 7, 2016, SLT is professional qualified to work with dysphagia. The present study observed comments that had in their content words and phrases such as “food refusal”, “dysphagia”, “diet”, “thickener”, “ingesting”, “feeding” in the exemplification of reasons for the use of TL in clinical practice. In this way, TL can be associated with SLT intervention as a way of guaranteeing diet intake and collaborating with the patient's adherence to the therapy.

SLTs needed to consider how ethical it would be to use TL as a communication strategy with people with dementia, also how valid it would be to lie to the patient to benefit them and to reduce caregivers' stress and how appropriate they considered the use of TL as a communication strategy. The majority of participants judged it as relatively ethical (77.8%), valid (65.3%) and appropriate (68.1%). However, the ethical question had the highest number of negative responses (9.7% - not ethical at all) when compared to validity (6.9% - not valid at all), appropriateness (8.3% - not appropriate at all), which shows the need addressed by previous studies^(12,18)^ for specific regulations so that professionals can apply the technique with greater mastery and safety.

Considering the lack of adequate instruction and documentation regarding the use of TL in the care of people with dementia that some studies have pointed out^(12,18)^, recommendations of organizations that are a reference in the subject of dementia were verified. The Alzheimer's Society of the USA (apud Culley et al.^(12)^) disapproves the use of TL to avoid inappropriate behaviour, while the Brazilian Alzheimer's Association (ABRAZ)^(24)^, which has a tab on its website with guidelines for dealing with symptoms of disease, does not mention the use of this strategy. Likewise, the code of ethics of SLT updated by the Brazilian Federal Council of Speech Therapy in 2021^(25)^, states in Chapter IV, Art. 7, item III: “making false statements about any situations or circumstances of SLT practice” is an ethical violation”. The above statement reflects on whether the use of TL by SLT is appropriate. Considering that the purpose of the technique is for the benefit of the patient, the use of TL is, at the same time, in line with Chapter IV, Art. 6, which deals with the professional's responsibilities and mentions that it is a duty of care for the SLT “to perform the practice fully, using all knowledge and resources needed to promote the well-being of the client”.

Ian James et al.^(18)^ addressed the creation and application of British guidelines, a 12 item set of recommendations on the use of TL. These guidelines are based on the principle that TL could only be considered as a form of therapy after verifying the cognitive abilities of each patient and verifying their inability to understand information given by professionals/caregivers and to make decisions for his/her best interest.

Thereby, the opinion of people with dementia must still be considered, as in the study by Day et al.^(14)^ carried out in the USA with this population participating in a discussion group, in which a questionnaire was applied about how acceptable they would consider being lied to. The results showed that patients believe that lying is valid only in certain circumstances, especially to ensure their safety or to minimise “truth-related stresses”, which indicates that individuals with dementia believe that the benefit from TL as a communication strategy is valid and effective.

Regarding the reports of situations in which SLT have used or could use TL as a communication strategy, it is possible to realise that most of the positive answers from the participants were focused on the patient's well-being, safety and collaboration. The negative answers showed a concern with ethical and practical aspects, with deontological opinions affirming that in their opinion lying is immoral, without considering the positive effects that the use of this strategy could have on the patient.

Furthermore, the results of the present study highlighted the lack of knowledge about TL, due to the shortage of bibliography and national research, in addition to the topic not being addressed during professional training. This is evident by the fact that 65.3% of the sample, stated that they have already used the strategy. However, only 23.8% had heard about it and only 5.6% had already found information on the topic in the literature. Possibly, these numbers are due to the fact that the technique is in regular use, but its users are not aware that it is a strategy, that with greater knowledge can have very positive effects in clinical practice. Noticing the number of SLTs who responded that were interested in knowing more about the subject, we suggest that further research be carried out, taking into account the opinion of Brazilian people with dementia and their relatives/caregivers. Simultaneously, this should be aligned with the development of recommendations for the use of TL, as in the protocol suggested by James et al.^(18)^ and/or validation of new protocols that guarantee the best way to use this strategy.

The present study presents a limitation regarding the sample. Since there is no real data on how many SLTs work in the field of dementia in Brazil, there is no reference to the distribution of the number of participants by regions, which makes it impossible to carry out a sample or statistical calculation on their representation. Possibly, the predominance of participants from the Southern region of the country was due to the fact that the researchers are from this region and have disseminated the research in their networks, which cover more professionals from this place. In this way, it can be considered a sampling bias.

## CONCLUSION

In general, Brazilian SLTs use TL in their clinical practice, taking into account the benefit of the person with dementia, although they recognize the lack of knowledge on this subject. Most participants considered this strategy relatively ethical, valid and appropriate, reinforcing the need for a regulation of its use to ensure the safety of those involved. Furthermore, the participants showed a real interest in learning more about TL, especially due to the lack of literature and contact with the technique during their professional training.

## Annex A. Questionnaire

1. Are you a Speech and Language Therapist?

() Yes () No

2. Are you Brazilian?

() Yes () No

3. Do you see patients with dementia?

() Yes () No

4. How old are you?

5. What gender do you identify with?

() Male () Female () Other

6. What is your highest educational level?

() Undergraduate () Graduate () Master () PhD () Post-doc

7. How many years have you been a Speech and Language Therapist?

8. How many years of experience do you have with patients with dementia?

9. What state of Brazil do you work in?

10. Have you ever lied to a patient to benefit her/him?

() Yes () No

10.1 If yes, in what situation did you lie or for what reason?

11. Have you ever guided relatives or caregivers of people with dementia on the use of therapeutic lying as a communication strategy?

() Yes () No

11.1 If yes, in what situation did you guide or for what reason?

12. Have you ever heard about the term “therapeutic lying”?

() Yes () No

13. Have you ever found bibliographic references on therapeutic lying?

() Yes () No

14. Have you been taught about therapeutic lying during your training?

() Yes () No

15. Would you like to learn more about therapeutic lying?

() Yes () No

16. How valid do you think it is to lie to a patient to benefit them and reduce the caregiver’s stress?

() Not valid at all () Relatively valid () Very valid

16.1 Would you like to comment on your choice?

17. How ethical do you think it is to use therapeutic lying as a communicative strategy to deal with challenging behaviours with people with dementia?

() Not ethical at all () Relatively ethical () Very ethical

17.1 Would you like to comment on your choice?

18. How appropriate do you think it is for a Speech and Language Therapist to use therapeutic lying as a communication strategy among caregivers/relatives of people with dementia?

() Not appropriate at all () Relatively appropriate () Very appropriate

19. In which of these situations do you think therapeutic lying could be used?

() To convince the patient to take the medications or receive treatments/health follow-ups

() To convince the patient to perform personal hygiene

() To convince the patient not to leave the house

() To convince the patient to eat well

() In no situation

() Others (comment below)

19.1 In which other situation could therapeutic lying be used?

20. Do you have comments, suggestions or criticisms?
