# Supplementary material for: Therapeutic lying: Brazilian speech and language therapists’ point of view about a controversial communication strategy employed in the care for people with dementia
Source: Codas. 2022 Oct 21;35(1):e20210252. doi: 10.1590/2317-1782/20212021252en (PMC10010423; doi:10.1590/2317-1782/20212021252en)
Supplement: Translation

# Translation: Mentira terapêutica: a visão do fonoaudiólogo brasileiro sobre uma estratégia de comunicação controversa no atendimento ao paciente com demência

**DOI:** 10.1590/2317-1782/20212021252pt

**Authors:** Isabella Amaral Lopes, Emily Viega Alves, Bárbara Costa Beber

**Affiliations:** 1 Curso de Fonoaudiologia, Universidade Federal de Ciências da Saúde de Porto Alegre – UFCSPA - Porto Alegre (RS), Brasil.; 2 Departamento de Fonoaudiologia, Universidade Federal de Ciências da Saúde de Porto Alegre – UFCSPA - Porto Alegre (RS), Brasil.; 3 Programa de Pós-graduação em Ciências da Reabilitação, Universidade Federal de Ciências da Saúde de Porto Alegre – UFCSPA - Porto Alegre (RS), Brasil.

**Keywords:** Demências, Comunicação, Fonoaudiologia, Decepção, Barreiras de Comunicação

## Abstract

**Objetivo:**

O objetivo deste estudo foi obter a visão do fonoaudiólogo brasileiro a respeito da utilização da mentira terapêutica como estratégia de comunicação no manejo de pacientes com demência.

**Método:**

Trata-se de uma pesquisa quantitativa e qualitativa, transversal, de caráter descritivo. A coleta de dados foi realizada de modo online através de um questionário contendo questões de múltipla escolha e resposta aberta.

**Resultados:**

Os resultados quantitativos indicaram que a maioria dos fonoaudiólogos já fizeram uso da estratégia e desejam aprender mais sobre, julgando a técnica como relativamente válida, ética e adequada. Os dados qualitativos indicaram os motivos para o uso da técnica, sendo estes: tranquilizar o paciente em situação de agitação; estimular o engajamento na terapia; evitar estresse relacionado à perda de memória; manejar dificuldade ou recusa alimentar; manejar dificuldade ou recusa ao tratamento medicamentoso; evitar que paciente evada o local; manejar situações de delírio, confusão e/ou paranoia; garantir a segurança; e quando outras estratégias não funcionam.

**Conclusão:**

A maioria dos fonoaudiólogos brasileiros utilizam a mentira terapêutica em sua prática clínica e o fazem levando em consideração o benefício da pessoa com demência, embora reconheçam a falta de conhecimento e preparo acerca do assunto. Consideraram essa estratégia de comunicação relativamente ética, válida e adequada. O artigo chama atenção para a necessidade de formação e de recomendações sobre o uso da mentira terapêutica entre pessoas com demência pelos fonoaudiólogos.

## INTRODUÇÃO

A demência é uma síndrome neurológica caracterizada por declínio cognitivo e funcional, com prejuízo em dois ou mais domínios cognitivos (como memória, funções executivas, atenção, linguagem, comportamento social e julgamento, capacidade psicomotora, entre outras) que não é relacionado ao envelhecimento natural^(1)^. É definida como uma síndrome adquirida de natureza crônica ou progressiva que interfere significativamente na independência da pessoa acometida^(2)^.

Dados da Organização Pan-Americana de Saúde de 2017^(3)^ indicam que o número de pessoas com demência poderá triplicar de 50 milhões para 152 milhões até 2050, conforme o envelhecimento da população mundial. No Brasil, diversos estudos^(4,5)^ demonstram a prevalência da doença por regiões com resultados variáveis. Os tipos mais frequentes de demência são a doença de Alzheimer (DA), demência vascular, demência de Corpos de Lewy, doença de Parkinson e demência frontotemporal^(4,5)^.

Pessoas com demência frequentemente apresentam alterações comportamentais que são difíceis de manejar, chamadas de comportamentos desafiadores^(6)^, que prejudicam a qualidade de vida desta população tanto fisicamente quanto psicologicamente, socialmente e economicamente. Estudos estimam que mais de 90% dos acometidos desenvolvem ao menos um comportamento desafiador no curso da doença^(7)^, sendo estes comportamentos os preditores da admissão destas pessoas em instituições de longa permanência^(8)^. Além disso, pessoas com demência também apresentam deterioração da linguagem, que pode causar uma redução na habilidade de manter conversações^(9)^ além dos déficits de memória ou de outros domínios cognitivos. Neste contexto, o fonoaudiólogo tem um papel fundamental na intervenção e tratamento desta população, pois promove a adaptação e treino de habilidades cognitivas, otimização das funções comunicativas e comportamentais, além de lançar mão do uso de estratégias de comunicação compensatórias a cada caso^(10)^.

Habilidades de comunicação são essenciais aos profissionais da sáude que atuam com pacientes sob cuidados paliativos, independente de sua formação ou área de especialidade^(11)^. Uma das estratégias de comunicação mais frequentemente empregadas para lidar com comportamentos desafiadores^(12-15)^ é a Mentira Terapêutica (MT) - uma mentira aplicada com o objetivo de atender o melhor interesse do paciente^(12)^, sobretudo quando há divergência entre a realidade do cuidador/profissional de saúde e da pessoa acometida pela doença^(16,17)^. Estudos apontam que a maioria dos profissionais de saúde já fez uso da MT na prática clínica^(6,13,18)^. A MT pode assumir um papel de intervenção e solução ágil para lidar com momentos críticos, pois representa um potencial de intervenção não-farmacológica no gerenciamento dos comportamentos desafiadores^(12)^. Ademais, poderia ser utilizada por fonoaudiólogos como estratégia de comunicação na terapia em casos em que a pessoa com demência é incapaz de compreender a informação recebida ou ação solicitada e tomar decisões para seu melhor interesse.

O que torna o uso dessa estratégia controverso é o fato de, geralmente, a mentira ser caracterizada, de acordo com Matias et al.^(19)^, como o ato de “tentar convencer outra pessoa a aceitar aquilo que o próprio indivíduo sabe que é falso, em benefício próprio ou de outros, para maximizar um ganho ou evitar uma perda” constituindo uma interação de valor social negativo, pois geralmente é utilizada para manipular, enganar, iludir ou distrair o outro^(13)^. Essas características põem em questão se a MT seria condizente com valores morais e éticos, principalmente no âmbito profissional. Há ainda a possibilidade de a MT ser considerada como uma forma de abuso à autonomia da pessoa com demência caso não seja regulamentada ou seja empregada de forma inadequada e ineficiente, pois é necessário saber definir quando a pessoa com demência é capaz de decidir por si mesma e como aplicar a estratégia de forma eficaz^(12)^.

Ao considerarmos a MT como uma estratégia de comunicação, o fonoaudiólogo, que é o profissional da saúde que atua em todos aspectos referentes à comunicação humana, poderia ter um papel fundamental na orientação quanto ao uso da MT por cuidadores e outros profissionais da saúde. No entanto, não há relatos na literatura de atuações e pesquisas da fonoaudiologia sobre o tema. Para que o fonoaudiólogo possa contribuir para a orientação do uso da MT é necessário primeiro saber se esta classe profissional conhece essa estratégia de comunicação e qual a sua opinião sobre o uso dela do ponto de vista ético. Apesar de estar ganhando mais atenção recentemente^(6,20-22)^, ainda há poucos estudos sobre o uso da MT como estratégia de comunicação no cuidado de pessoas com demência, possuindo pouca evidência científica mesmo sendo amplamente utilizada na prática clínica. Torna-se imprescindível informar sobre a estratégia e habilitar fonoaudiólogos (e esses, por conseguinte, os cuidadores e familiares) a intervir apropriadamente nas situações em que pacientes apresentam comportamentos desafiadores, visando sempre o melhor interesse do paciente. Dessa forma, o objetivo do presente estudo é obter a visão do fonoaudiólogo brasileiro a respeito da utilização da MT como estratégia de comunicação no manejo de pacientes com demência.

## MÉTODO

Trata-se de uma pesquisa quantitativa e qualitativa, transversal, de caráter descritivo, que foi aprovada pelo comitê de ética local sob parecer de número 2.733.408. A amostra deste estudo se caracteriza como uma amostra de conveniência.

A coleta de dados foi realizada de modo online através de um questionário (Anexo A) elaborado pelas pesquisadoras, contendo 25 questões, sendo 14 de múltipla escolha e 11 de resposta aberta, direcionado a fonoaudiólogos do Brasil. O questionário foi elaborado pelas autoras e disponibilizado na plataforma *Google Forms* para que os participantes pudessem responder individualmente. O acesso às questões foi permitido somente após a confirmação de leitura e concordância dos participantes referente ao Termo de Consentimento Livre e Esclarecido (TCLE), disponibilizado de forma online previamente à apresentação do questionário. Para consentir em participar do estudo, o participante precisava clicar em “eu concordo”.

O estudo foi divulgado através de redes sociais e de listas de e-mails. Foram incluídos fonoaudiólogos nascidos e atuantes no Brasil com experiência na área de demências. Estudantes de fonoaudiologia, fonoaudiólogos aposentados ou não atuantes em demências foram considerados como critérios de exclusão. Os participantes que não concluíram o questionário foram excluídos do estudo.

Os dados foram exportados da plataforma Google Forms para Google SpreadSheets e transformados em *Microsoft Excel* para serem analisados posteriormente através do programa *Statistical Package for the Social Sciences* (SPSS), versão 21. Quanto aos dados quantitativos, as variáveis contínuas foram descritas em média e desvio padrão, e as categóricas em frequência absoluta e relativa. Os dados qualitativos foram analisados através do método de análise de conteúdo^(23)^, sumarizados pelas autoras e descritos em forma de narrativa.

## RESULTADOS

### Resultados quantitativos

Responderam ao questionário 82 sujeitos. Destes, um foi excluído por não ser fonoaudiólogo e nove por não possuírem experiência com pessoas com demência. Sendo assim, a amostra final foi composta por 72 participantes.

Em relação aos dados descritivos, evidenciou-se o predomínio do sexo feminino (100%). A especialização (48,6%) foi maioria quanto ao grau de escolaridade, seguida por doutorado (18,1%), mestrado (13,9%), graduação (12,5%) e pós-doutorado (6,9%). A região sul foi observada como região demográfica mais frequente (45,8%) seguida pela região sudeste (33,3%), centro-oeste (12,5%), nordeste (6,9%) e norte (1,4%). O tempo de formação desses profissionais teve como média 12,51 anos (±9,89), enquanto o tempo de experiência com demência 7,56 anos (±5,97).

Quanto à opinião dos fonoaudiólogos sobre o uso da MT, 77,8% da amostra considera a prática relativamente ética, 12,5% considera muito ética e 9,7% não considera nem um pouco ética. Quando perguntados sobre o quão válido é mentir para beneficiar o paciente e reduzir o estresse dos cuidadores, 65,3% consideraram relativamente válido, enquanto 27,8% consideraram muito válido e 6,9% nem um pouco válido. Por fim, em relação à opinião da amostra sobre o uso da MT pela classe profissional, 68,1% dos participantes manifestaram que a prática é relativamente adequada, 23,6% manifestaram que ser muito adequada e 8,3% consideraram que não é nem um pouco adequada.

A Tabela 1 diz respeito ao uso e conhecimento da MT pelos fonoaudiólogos participantes do estudo, enquanto a Tabela 2 representa situações em que a MT poderia ser utilizada pela ótica dos participantes.

**Tabela 1:** Uso e conhecimento da MT pelos fonoaudiólogos participantes do estudo (frequências referentes às respostas “sim”)

	**N (%)**
Você já mentiu para um paciente com demência para beneficiá-lo?	47 (65,3)
Você já orientou familiares ou cuidadores de pessoas com demência sobre o uso da MT como estratégia terapêutica?	35 (48,6)
Você já havia ouvido falar no termo MT antes desta pesquisa?	17 (23,6)
Você já encontrou referências bibliográficas sobre a MT?	4 (5,6)
Você foi ensinado sobre a MT durante a sua formação?	6 (8,3)
Você gostaria de aprender mais sobre a MT?	59 (81,9)

Legenda: MT = mentira terapêutica

**Tabela 2:** Situações em que a mentira terapêutica poderia ser utilizada

	N (%)
Para convencer o paciente a tomar as medicações ou realizar tratamentos/acompanhamentos de saúde	53 (82,8)
Para convencer o paciente a realizar a higiene pessoal	46 (71,9)
Para convencer o paciente a não sair de casa	40 (62,5)
Para convencer o paciente a se alimentar adequadamente	51 (79,7)
Em nenhuma situação	7 (10,9)
Outra (comente na pergunta a seguir)	10 (15,6)

### Resultados qualitativos

Quanto aos dados qualitativos, foram elaboradas perguntas de resposta aberta para que os participantes pudessem comentar suas escolhas com relação aos motivos e situações que os levaram a utilizar a MT como estratégia na sua prática clínica. Para realizar a análise desses dados, utilizou-se o método de análise de conteúdo^(23)^. Alguns exemplos de relatos dos participantes foram agrupados nas categorias expostas no Quadro 1.

**Quadro 1:** Dados qualitativos

Categorias de motivos para o uso da MT	Exemplos de relatos
Tranquilizar em situação de agitação	Profissional informou ao paciente que seu acompanhante estava na sala de espera, mesmo não estando, apenas com o intuito de tranquilizá-lo.
Colaboração com a terapia	Fonoaudiólogo mencionou que a consulta do paciente era paga pelo plano de saúde, quando na verdade os filhos o faziam.
Evitar estresse relacionado à perda de memória	MT utilizada para evitar sofrimento dos pacientes que não lembram do falecimento de seus cônjuges ou entes queridos.
Dificuldade ou recusa alimentar	Profissional relatou que um paciente só aceitava a dieta quando lhe era dito que havia sido preparada pela mãe ou tia, ambas falecidas.
Dificuldade ou recusa ao tratamento medicamentoso	Sujeito informou trabalho em conjunto com médico para encontrar formas de minimizar o estresse na administração do medicamento.
Evitar que paciente evada o local	MT empregada para evitar que o paciente se frustre ou se agite pela impossibilidade de evadir locais.
Lidar com delírio, confusão e/ou paranoia	Fonoaudióloga recorreu a MT ao confirmar que era uma enfermeira e que cuidaria dos ferimentos de guerra do paciente.
Garantir a segurança	Profissional experienciou uma paciente que não deambulava e queria levantar-se da cadeira para sair de casa, utilizando a MT para dissuadi-la da situação.
Quando outras estratégias não funcionam	MT foi o único recurso eficaz quando a mudança de foco de atenção do paciente não foi efetiva.

A respeito do quão válido e ético seria o uso da MT, dividiu-se os comentários correspondentes a cada item da escala Likert de três pontos (Muito, Relativamente e Nem um pouco) em cada pergunta.

Quando questionados sobre o quão válido acreditavam que seja mentir para o paciente para beneficiá-lo, aqueles que consideraram a prática como “muito válida”, defenderam a MT para garantir a cooperação, tranquilidade e segurança do paciente de forma rápida. Os fonoaudiólogos que defenderam esta opção reforçaram a importância da autorização da família. Os sujeitos que optaram por responder “relativamente válido” afirmaram que é necessário averiguar o momento adequado e o conteúdo utilizado. Estes profissionais consideram o uso da MT como último recurso, aplicando primeiramente outras estratégias, como a utilização de temas distratores para mudança de foco. Os fonoaudiólogos que assinalaram a opção “nem um pouco válido” expressaram que a mentira constitui um engano e fere as relações interpessoais, e os benefícios seriam mais voltados aos familiares e cuidadores. Nesse sentido, eles acreditam que outras técnicas desempenham melhor o papel que a mentira teria no cuidado das pessoas com demências.

Em relação ao quão ético os sujeitos consideraram a MT, os que marcaram a opção “muito ético” alegaram que a intenção da MT não é enganar ou esconder algo do paciente, mas sim de manejar o comportamento que ele apresenta para o seu próprio bem. Os sujeitos que consideraram o uso da MT como “relativamente ético” justificaram sua escolha através da autonomia do paciente como foco e da qualidade da informação utilizada no emprego da estratégia, julgando a participação da família como fundamental. Ao selecionar a opção “nem um pouco ético”, a motivação apresentada pelos participantes baseou-se principalmente na definição de ética, visto que, para eles, a mentira caracteriza uma quebra deste princípio.

No que concerne aos comentários sobre outras situações em que a MT poderia ser utilizada, considerou-se apenas as respostas que não haviam sido previamente mencionadas em outros itens do questionário. A única questão destacada nesta seção foi a necessidade de um conhecimento maior acerca da MT como estratégia de comunicação.

## DISCUSSÃO

O presente estudo investigou a visão do fonoaudiólogo brasileiro sobre o uso da MT como uma estratégia de comunicação no manejo de pacientes com demência, visto que a fonoaudiologia é a profissão que tem como objeto de estudo a comunicação humana. A atuação do fonoaudiólogo neste tema poderia se dar através do uso da MT como uma estratégia de comunicação durante seu atendimento, a fim de minimizar alterações comportamentais dos pacientes e garantir uma melhor adesão dos mesmos, assim como, através do treinamento e orientação de cuidadores e outros profissionais da saúde sobre como usar a MT adequadamente nesta população.

Os achados do presente estudo corroboram com os resultados das pesquisas previamente feitas com outros profissionais de saúde, como psicólogos, psiquiatras e enfermeiros^(12,15)^, em relação ao fato destes profissionais já terem utilizado a MT na sua prática clínica, mesmo que não estivessem apropriadamente informados sobre a estratégia de comunicação e suas questões éticas.

O quadro 1 revelou as diversas situações em que os participantes utilizaram a MT. Evidenciou-se que, em todas as situações, o objetivo foi atender o melhor interesse do paciente. Dentre as diversas categorias expostas, chama-se atenção para o tópico “dificuldade ou recusa alimentar”. Segundo a Resolução do Conselho Federal de Fonoaudiologia, nº 492, de 7 abril de 2016, o fonoaudiólogo é o profissional habilitado na atuação em disfagia. O presente estudo observou comentários que possuíam em seu conteúdo palavras e frases como “recusa alimentar”, “disfagia”, “dieta”, “espessante”, “ingerir” e “alimentação” na exemplificação de motivos para o uso da MT na prática clínica. Dessa forma, a MT pode ser aliada como forma de garantir a ingestão da dieta e colaborar com a adesão do paciente à intervenção fonoaudiológica.

Quando os fonoaudiólogos precisaram considerar o quão ético seria utilizar a MT como estratégia de comunicação com pessoas com demência, o quão válido seria mentir para o paciente para beneficiá-lo e para reduzir o estresse dos cuidadores, além do quão adequado consideram a utilização da MT como estratégia de comunicação a ser trabalhada com familiares/cuidadores de pessoas com demência pelo profissional da fonoaudiologia, a grande maioria julgou como relativamente ético (77,8%), válido (65,3%) e adequado (68,1%). No entanto, a questão ética apresentou o maior número de respostas negativas (9,7% - nenhum pouco ético) quando comparada à validez (6,9% - nenhum pouco válido) e adequação (8,3% - nenhum pouco adequado), evidenciando a necessidade abordada por estudos prévios^(12,18)^ de uma regulamentação específica de referência para que os profissionais possam aplicar essa técnica com maior propriedade e segurança.

Considerando a carência de instrução e documentação adequada quanto a utilização de MT na assistência a pessoas com demência que algumas pesquisas^(12,18)^ apontam, verificou-se as recomendações de organizações que são referência no tema das demências a para a profissão da fonoaudiologia. A sociedade de Alzheimer dos EUA (apud Culley et al.^(12)^) condena o uso da MT para evitar estresse, enquanto a Associação Brasileira de Alzheimer (ABRAZ)^(24)^, que possui em seu website uma aba com diversas orientações para lidar com os sintomas da doença, não faz menção ao uso dessa estratégia em particular. Do mesmo modo, verificou-se o código de ética da fonoaudiologia atualizado pelo Conselho Federal de Fonoaudiologia em 2021^(25)^, cujo Cap. IV, Art. 7º, item III descreve que: “fazer declarações falsas sobre quaisquer situações ou circunstâncias da prática fonoaudiológica” consiste em infração ética. Este item do código de ética traz a reflexão sobre o uso da MT pelo profissional fonoaudiólogo ser apropriado. Considerando que o objetivo da técnica é o benefício do paciente, o uso da MT vai, ao mesmo tempo, ao encontro do Cap. IV, Art. 6º, que trata das responsabilidades do profissional e menciona que é um dever do fonoaudiólogo “exercer a atividade de forma plena, utilizando-se dos conhecimentos e recursos necessários, para promover o bem-estar do cliente”.

O estudo de James et al.^(18)^ aborda a criação e aplicação de um protocolo de orientações, composto por um conjunto de 12 diretrizes sobre o uso da MT. Estas diretrizes têm como princípio que a MT só poderia ser considerada como forma de terapia após a verificação das habilidades cognitivas de cada paciente e constatação da incapacidade deste de compreender informações dadas pelos profissionais/cuidadores e de tomar decisões para seu melhor interesse.

Tendo isso em vista, há ainda de se considerar a opinião das pessoas acometidas pela doença, como no estudo de Day et al.^(14)^ realizado nos EUA com pessoas com demência participantes de um grupo de discussão, em que um questionário foi aplicado sobre o quão aceitável eles considerariam que fosse mentido para eles. Obteve-se resultados que demonstraram que pacientes acreditam que a mentira é válida somente em determinadas circunstâncias, especialmente para garantir a segurança ou para minimizar “estresses relacionados à verdade”, o que indica que esta população acredita beneficiar-se da MT como estratégia de comunicação válida e eficaz.

Sobre os relatos de situações em que os fonoaudiólogos utilizaram ou poderiam utilizar a MT como estratégia de comunicação, é possível perceber que a maior parte das respostas positivas focou no bem-estar do paciente como motivo principal, além da segurança e colaboração do mesmo. Já as respostas negativas demonstraram uma maior preocupação com os aspectos éticos e práticos, contando com opiniões deontológicas afirmando que a mentira é imoral, sem considerar os efeitos positivos que o uso dessa estratégia acarretaria para o paciente.

Ademais, os resultados do presente estudo salientaram a falta de conhecimento sobre a MT, devido à escassez de bibliografia e pesquisas nacionais, além do tema não ser abordado durante a formação profissional. Isso se mostra evidente pelo fato de que 65,3% da presente amostra ter afirmado que já utilizou a estratégia, no entanto, apenas 23,8% já ouviram falar sobre ela e apenas 5,6% já encontraram informações sobre o tema na literatura. Possivelmente, esses números se devem ao fato de que a técnica é de uso regular, porém seus usuários não têm conhecimento que se trata de uma estratégia que, com maiores conhecimentos, pode ter grandes efeitos positivos na prática clínica. Atentando para a quantidade de fonoaudiólogos que responderam ter interesse em saber mais sobre o assunto, sugere-se que mais pesquisas sejam feitas levando em consideração a opinião de pessoas brasileiras com demência e seus familiares/cuidadores, conjuntamente com o desenvolvimento de recomendações para o uso da MT, a exemplo do protocolo sugerido por James et al.^(18)^, e/ou validação de novos protocolos que garantam a melhor forma de fazer uso dessa estratégia.

O estudo realizado apresentou uma limitação em relação à amostra, que deve ser levada em conta ao interpretar os resultados. Visto que não há dados reais sobre quantos fonoaudiólogos atuam na área de demências no Brasil, não há referência da distribuição de número de participantes pelas regiões, o que impossibilitou um cálculo amostral ou estatístico sobre a representatividade delas na amostra. Possivelmente, houve predomínio de participantes do sul do país devido ao fato de as pesquisadoras serem desta região e terem divulgado a pesquisa em suas redes, que abrangem mais profissionais deste local, o que pode ser considerado, desta forma, um viés amostral.

## CONCLUSÃO

De modo geral, os fonoaudiólogos brasileiros utilizam a MT em sua prática clínica e o fazem levando em consideração o benefício da pessoa com demência, embora reconheçam a falta de conhecimento acerca do assunto. A grande maioria dos participantes considera essa estratégia de comunicação relativamente ética, válida e adequada, reforçando a necessidade de regulamentação que supervisione seu uso para garantir a segurança dos envolvidos e demonstrando um grande interesse em aprender mais sobre, dada a escassez de literatura e contato com a técnica durante a formação profissional.

## Anexo A. Perguntas do questionário

1. Você já é um(a) fonoaudiólogo(a) formado(a)?

() Sim () Não

2. Você é brasileiro(a)?

() Sim () Não

3. Você atende pacientes com demência?

() Sim () Não

4. Qual a sua idade?

5. Você se identifica com que gênero?

() Masculino () Feminino () Outro

6. Qual a sua escolaridade?

() Graduação () Pós-graduação () Mestrado () Doutorado () Pós-doutorado

7. Quantos anos de formação em fonoaudiologia você possui?

8. Quantos anos de experiência com demências você possui?

9. Em qual estado você atua?

10. Você já mentiu para um paciente para beneficiá-lo?

() Sim () Não

10.1 Se sim, em qual situação você mentiu ou por qual motivo?

11. Você já orientou familiares ou cuidadores de pacientes com demência sobre o uso da mentira como estratégia comunicativa?

() Sim () Não

11.1 Se sim, em qual situação você orientou ou por qual motivo?

12. Você já ouviu falar no termo “mentira terapêutica”?

() Sim () Não

13.Você já encontrou referências bibliográficas sobre a mentira terapêutica?

() Sim () Não

14.Você foi ensinado(a) sobre a mentira terapêutica na sua formação?

() Sim () Não

15.Você gostaria de aprender mais sobre esta técnica?

() Sim () Não () Talvez

16. O quão válido você acha que é mentir para o paciente para beneficiá-lo e reduzir o estresse dos cuidadores?

() Nenhum pouco válido () Relativamente válido () Muito válido

16.1 Você gostaria de comentar a sua escolha?

17. O quão ético você acha que é utilizar a mentira terapêutica como estratégia comunicativa para o manejo de comportamentos difíceis em pessoas com demência?

() Nenhum pouco ético () Relativamente ético () Muito ético

17.1 Você gostaria de comentar a sua escolha?

18. O quão adequado você acha que é o fonoaudiólogo utilizar a mentira terapêutica como uma estratégia de comunicação a ser trabalhada com cuidadores/familiares de pessoas com demência?

() Nenhum pouco adequado () Relativamente adequado

() Muito adequado

19. Em quais dessas situações você acha que a mentira terapêutica poderia ser utilizada?

() Para convencer o paciente a tomar medicações ou realizar tratamentos/acompanhamentos

() Para convencer o paciente a realizar higiene pessoal

() Para convencer o paciente a não sair de casa

() Para convencer o paciente a se alimentar adequadamente

() Em nenhuma situação

() Outra (comente na pergunta a seguir)

19.1 Em qual outra situação a mentira terapêutica poderia ser utilizada?

20. Comentários, sugestões, críticas?
